# Emotional Health of Immigrant Adolescents by a Cross-Lagged Panel Network Analysis: Self-Esteem and Depression

**DOI:** 10.3390/healthcare12242563

**Published:** 2024-12-19

**Authors:** Tiange Sui, Jerf W. K. Yeung

**Affiliations:** 1 Department of Social and Behavioral Sciences, City University of Hong Kong, Kowloon, Hong Kong, China; 2 Graduate School of Human Sciences, Osaka University, Osaka 565-0871, Japan; ssjerf@gmail.com

**Keywords:** self-esteem, depression, immigrant adolescents, cross-lagged panel network (CLPN), mental health care

## Abstract

**Background/Objectives**: The study investigated the dynamic interrelations of both positive and negative self-esteem with depression among immigrant adolescents. **Methods**: Longitudinal data from the Children of Immigrants Longitudinal Study (CILS) were analyzed using a Cross-Lagged Panel Network (CLPN) model. **Results**: The results showed strong autoregressive effects; both the positive and negative dimensions of self-esteem and symptoms of depression were fairly stable across the two measurement times. Cross-lagged effects indicated that higher levels of positive self-esteem predicted reduced depressive symptoms; for example, higher self-worth at Time 1 was associated with a lower lack of motivation at Time 2. However, some components, for instance, positive self-attitude, predicted in greater sadness from Time 1 to Time 2. On the other hand, certain dimensions of negative self-esteem, such as feeling useless at Time 1, were related to decreases in depressive symptoms at Time 2, which points to complex and bidirectional effects that challenge traditional hypotheses on how self-esteem may affect mental health. **Conclusions**: The current study teases apart sub-components of self-esteem and, in doing so, demonstrates how different facets uniquely predict depression over time and inform nuanced mental health trajectories among immigrant youth. The findings indicate that selective self-esteem interventions should be carried out to enhance resilience and mental well-being in adolescents from diverse backgrounds.

## 1. Introduction

The development of self-esteem during adolescence plays a critical role in shaping long-term mental health and behavioral outcomes that impose long-term impacts on the later physical, psychological, and social well-being of adolescents in adulthood throughout their life trajectories [1,2]. Self-esteem, or the overall sense of self-worth or personal value, dramatically changes during the formative stage of adolescence and is highly influenced by external factors, such as peer relationships, family dynamics, and societal experiences [1,2]. These changes are even more striking and require even more attention among minority groups of a population, such as immigrant and ethnic-minority adolescents, who are more susceptible to the experiences of discrimination and social exclusion, which are believed to have direct relevance to the etiology of depression, an important dimension of emotional health [3,4,5]. Seaton, Neblett, Cole and Prinstein [6] reported that immigrant adolescents experiencing higher levels of discrimination are more likely to report symptoms of depression, which would lead to a vicious cycle where low self-esteem and depressive symptoms reinforce each other [7]. Despite a great number of studies investigating the association between self-esteem and depression [8,9], less is known about the temporal dynamics of these constructs—that is, how changes in these constructs affect each other.

Although most traditional models viewed self-esteem as a unidimensional construct [10], there is growing evidence to suggest that the underlying processes involved in multidimensional functioning—that is, positive and negative self-esteem-take different routes and may carry different implications for psychological well-being [1,11]. For example, positive self-esteem—that is, a representation of self-worth [5] and competence—may buffer against negative emotional health outcomes, while negative self-esteem—that is, feelings of failure and worthlessness—may amplify this risk for symptoms of depression [12]. The aforementioned dimensions of self-esteem were considered high-priority [12] because their time-lagged effects are crucial in capturing the dynamic interplay between self-esteem and depression during adolescence.

Recent studies have emphasized the need for longitudinal approaches that tease apart the directionality of the self-esteem and depression association [13,14]. Although it is well-established that low self-esteem is associated with higher levels of depression [8,13], the temporal dynamics in this relationship are not as explored. Concretely, limited studies have investigated how positive and negative self-esteem differently predict future depressive symptoms. In turn, depressive symptoms might also predict subsequent multiple dimensions of self-esteem over time. While positive self-esteem may perhaps guard against depression for a greater length of time [15], negative self-esteem is able to predict an increase in depressive symptoms over time [16]. In return, depression further lowers negative self-esteem, forming a self-enhancing cycle with time [9].

For this reason, the present study aims to examine the dynamic time-lagged relationships between positivity/negativity of self-esteem and depression in immigrant adolescents. Based on a Cross-Lagged Panel Network (CLPN), within a two-point measurement timeline design, the study plans to investigate the cross-lagged effects of self-esteem and depression. More specifically, we will explore whether positive self-esteem predicts a decrease in future depressive symptoms, and whether negative self-esteem predicts an increase in such symptoms among the adolescent immigrants. We also explored the degree to which depression influences self-esteem over time.

### 1.1. Self-Esteem Dimensions in Immigrant Adolescent Development

Various articles refer to self-esteem as an element of basic psychological well-being [1], especially when considering the development of self in relation to identity during adolescence [17], in which the need for acquisition during adolescent development from peers and adults alike becomes stronger; thus, self-esteem becomes a very significant ingredient in how they are going to feel socially and emotionally [18]. More than that, besides the general problems pertinent to all adolescents, an immigrant adolescent has to face a set of particular stressors connected with cultural adaptation and development of identities [19]. Indeed, a large proportion of the immigrant youths go through acculturative stress—a result of the conflicting desires to learn a new culture while at the same time keeping allegiance to their culture of origin [4]. Cultural inevitability occurs when people from different cultures come into contact with each other [20]. This process of change is often stressful and has been found to have negative consequences for self-esteem, especially when adolescents are made to feel like marginal members of society on the basis of their ethnicity [3,6]. In these cases, good self-esteem seems to act as a protective factor in the face of critical negative externals that establish stressors for the adolescent immigrant in maintaining a positive sense of self-worth [21]. The latter negative self-esteem state is characterized by feelings of failure or inadequacy, which only increase the psychological problems when struggling to find one’s way through these dual cultural identities [19].

The theoretical literature of the self-concept model explained by Harter [22] or Rosenberg’s self-esteem scale [23] provides necessary insights to support that the concept of self-esteem should be considered dynamic rather than static [13] and it is susceptible to being influenced by external factors [2,24]. For immigrant adolescents, such influences may be even stronger and may occur in addition to dealing with problems related to peer popularity, language barriers, and financial difficulties [25]. For instance, common self-esteem develops within an interactive process of social relationships of acceptance and refusal by peers and in a broader cultural context [26]. Accordingly, it is bolstered through positive reinforcement from friends, teachers, and family, while it is lessened through negative experiences such as discrimination or social exclusion [1]. Thus, a balance between the supportive experience promoting a positive self-concept and adversities challenging one’s sense of belonging/self-worth determines the dimensions of the self-esteem for immigrant adolescents [12,27].

Recent research has underlined a multidimensional view of self-esteem, including both the positive and negative dimensions of self-esteem [12,27,28]. Whereas all the dimensions of positive self-esteem protect against the development of psychiatric problems, negative self-esteem is considered a major risk factor for the development of depression [5,7]. The distinction between the two dimensions is even clearer in immigrant populations, whose ability to reconcile their dual identities and handle the challenges of acculturation impacts psychological well-being in a very strong manner. Nguyen and Benet-Martínez [29] present evidence as to how these two dimensions interact with depression over time, especially through longitudinal approaches that detail specific mechanisms driving those relationships. On one hand, for instance, positive self-esteem may protectively act against the development of depressive symptoms within a context of discrimination, while negative self-esteem acts as a vulnerability factor, increasing the psychological weight of such stressors [9,28,30]. Most especially in immigrant adolescents, positive self-esteem could heighten cultural pride and resilience against discriminatory pressures coming from the larger society. A strong sense of ethnic pride concurrently with a feeling of competent ability to handle the mainstream culture usually characterizes adolescents showing higher levels of well-being and, in general, psychological adjustment [30]. On the other hand, negative self-esteem (self-criticism, feelings of failing, and worthless) has been associated with higher levels of psychological distress, even encompassing anxiety, depression, and suicidal ideation [31,32]. Negative self-esteem may also lead an immigrant adolescent to internalizing disorders such as depression, especially when one fails to satisfy an expectation from either their home culture or the host society [4]. This is further compounded by an added layer of cultural adjustment stress that may heighten vulnerability to negative self-perceptions. In summary, self-esteem is central to the emotional health of immigrant adolescents and has been influenced by many social and cultural variables [1]. While it is positive self-esteem that nurtures pride in culture and resilience, negative self-esteem increases the risk of mental disorders [10]. Understanding and supporting the multidimensional nature of self-esteem in immigrant adolescents is thus critical to better their overall psychological adjustment.

### 1.2. Positive and Negative Self-Esteem and Depression

The critical terms in understanding mental health during adolescence are how these two self-esteem components interact with depression [8]. Numerous findings suggesting a strong connection between low self-esteem and increased depressive symptoms [8,33]. In the case of immigrant adolescents, positive self-esteem is particularly important, as they have to go through the acculturative stress—a result of psychological influence exerted by the necessity to adapt to a new culture while retaining ties with their heritage [34]. Statements such as “I have a number of good qualities” stimulate the development of such a self-concept that protects an individual from the negative influence of discrimination and social exclusion [35], which are so widespread in the lives of immigrant youth [36,37]. Consequently, it is important to establish that in adolescents, positive self-esteem allows them to withstand the social burdens and diminish feelings of alienation or inadequacy, thus decreasing the manifestation of depressive symptoms [38]. By internalizing positive self-beliefs, adolescents can foster resilience and reduce the impact of depressive symptoms that might otherwise result from acculturative pressures and identity conflicts [39].

By contrast, negative self-esteem items, like “I’m inclined to feel I’m a failure” and “I certainly feel useless at times”, may facilitate vulnerability to depressive symptoms due to specifically exacerbating feelings of sadness and worthlessness that arise during depression [7,40,41]. Various studies have pointed out that teenagers constantly visiting such statements are at a higher risk for internalizing disorders, which include depression and can develop symptoms of low energy, a lack of interest in daily activities, and pervasive sadness [31,42]. Whereas negative self-perceptions may undermine the protective effects of positive self-esteem, both dimensions of positive and negative self-esteem were considered critical in understanding depression outcomes for individuals [28,43].

In the process of cultural adjustment, self-esteem may be accentuated among immigrant adolescents due to the encountered discrimination and identity conflicts. These might feel that they do not belong to either their heritage culture or the host culture, adding to an exaggerated view of oneself as a failure or worthless [44]. Studies have shown that incidents of racial or ethnic discrimination heighten feelings of alienation and social isolation, which promote negative self-talk that further lowers self-esteem [29]. For instance, the item “I certainly feel useless at times” might strongly apply to immigrant youth who experience these struggles, in which rejected and excluded experiences reinforce internalized self-views of inadequacy [45,46]. Negative self-esteem can undermine motivation [47], and this decline in motivation is reflected in various symptoms. For instance, adolescents who view themselves as having “not much to be proud of” may have difficulty engaging in social or academic activities. Such disengagement may reduce their opportunities to find support networks and experiences positive reinforcement [42]. This in turn nurtures their negative self-concept, developing a vicious cycle that further increases depressive symptoms [33]. Thus, negative self-esteem is not only a contributor to immediate depressive symptoms but also instills such a cycle where low self-worth feeds into feelings of sadness, hopelessness, and lack of engagement in social activities.

### 1.3. Bidirectional Nature of Self-Esteem and Depression

While research consistently shows that negative self-esteem can increase the risk of depressive symptoms, emerging evidence suggests a bidirectional relationship between these constructs. Orth and Robins [1] found that low self-esteem not only predicts future depression but that depression itself can also lead to further declines in self-esteem, creating a reinforcing cycle. Such a situation may be considered particularly annoying for immigrant teenagers who already can face acculturative stress, identity conflict, and discrimination [7]. Longitudinal studies have further supported the bidirectional relationship between self-esteem and depression [9,13], proposing that changes in one construct often precede changes in the other over time [33]. For instance, depressive symptoms like persistent sadness and loss of motivation can erode self-worth, while low self-esteem may make adolescents more susceptible to the emotional and psychological impacts of social rejection and peer comparison [9]. This reciprocal pattern is especially relevant for immigrant adolescents, for whom the additional challenges of cultural adaptation can increase the likelihood of negative self-appraisals [39].

### 1.4. Current Study

Although there is an established relationship between self-esteem and depression, the dynamic-involving time-lagged effects of positive and negative self-esteem on depression-has yet to be mapped, especially among immigrant adolescents. By using a longitudinal design that targets dimensions of self-esteem, the current investigation focuses on the time-related predictive quality of positive versus negative self-esteem for depressive symptoms. Thereby, the present research intended to use the CLPN modeling procedures to analyze the bidirectional association between self-esteem and depression among immigrant adolescents across time for providing more dynamic empirical evidence about the development of emotional health of this adolescent group, which can help the design and implementation of more relevant and effective health care and mental health interventions and services appropriate for their needs. Specifically, as informed by multidimensional theories of self-esteem [28,43], the current study planned to inspect the impacts of positive and negative facets of self-esteem on specific depressive symptoms of immigrant adolescents, and vice versa, to clarify their distinctive effects on each other.

## 2. Materials and Methods

### 2.1. Study Design

The current study is of longitudinal design and aims to establish the interrelations of self-esteem and depression among immigrant adolescents at two critical points, with consideration for changes throughout adolescence. The sample population consisted of second-generation immigrant adolescents attending the 8th and 9th grade in public and private school, selected from the Children of Immigrants Longitudinal Study (CILS) in two phases [48]: the first time period was in 1991, during which participants were in early adolescence (12–14 years), and the second time period was in 1995, when the respondents reached late adolescence (16–18 years). The random sample included 5262 participants from 77 nationalities. The largest groups consisted of Cubans, Haitians, Nicaraguans, and West Indians in Florida, and Mexicans, Filipinos, Vietnamese, Laotians, and Cambodians in California.

The Cross-Lagged Panel Network (CLPN) analysis method was utilized in order to investigate the dynamic relationship between the variables. In the CLPN model [49], autoregressive effects (where a variable is predicted from its previous value over time) and the cross-lagged effects (where a variable at later time is predicted by another variable at an earlier time) were investigated. The main analyses controlled for the key confounding variables of gender, age, and socioeconomic status. This design allows an in-depth analysis of the developmental trajectories of self-esteem and depression in different dimensions, and how they may interact across cultural, familial, and socio-economic contexts in adolescence.

### 2.2. Measures

Self-Esteem was measured using a short form of the Rosenberg Self-Esteem Scale [40]. The items measuring positive self-esteem have: “I am a person of worth”, “I have a number of good qualities”, “I take a positive attitude toward myself”, and “I am satisfied with myself”. The items for negative self-esteem include: “I’m inclined to feel I’m a failure”, “I do not have much to be proud of”, “I certainly feel useless at times”, and “At times I think I am no good at all”. These items were chosen because they are theoretically important and empirically supported in prior research on self-esteem dimensions [1,50]. Such diversities have shown very good internal consistency across diverse populations, with Cronbach’s alpha usually ranging between 0.77 and 0.88 [50,51]. The internal consistency of self-esteem is acceptable reliability across both timelines. In T1, Cronbach’s alpha was 0.71 for positive self-esteem and 0.73 for negative self-esteem. In T2, positive self-esteem had an alpha of 0.74, and negative self-esteem was 0.74, supporting the reliability of these constructs over time.

Depression was measured using items from the CES-D Depression Scale provided in the CILS [52]. These items included: “Felt sad past week”, “Couldn’t get going past week”, “Didn’t feel like eating past week”, and “I felt depressed past week”. These selected items capture the core emotional characteristics of depression [53]. Generally, internal reliability for the CES-D was good, with Cronbach’s alpha often being above 0.85 across samples [52,53]. The internal consistency for depression items was acceptable, with Cronbach’s alpha of 0.73 at T1 and 0.76 at T2.

### 2.3. Data Analysis

The data analyses of the current study were conducted using R (version 4.4.1), invoking packages of lavaan, mice, qgraph, and bootnet [54,55]. This paper examines the dynamic interaction between self-esteem and depression across the two times periods; most importantly, it places great emphasis on the differentiating roles of positive and negative self-esteem in this process. The associations under scrutiny were primarily examined through the Cross-Lagged Panel Network CLPN model, which takes time-lagged effects and autoregressive paths between variables into account. To investigate the dynamic bidirectional relationships between self-esteem and depression over time, we applied the Cross-Lagged Panel Network (CLPN) modeling that was fitted in R programming software (version 4.3.2) using the lavaan package [56]. A CLPN model allows us to estimate both the autoregressive effects (how variables predict themselves over time) and the cross-lagged effects (how one variable at Time 1 predicts another variable at Time 2).

Before modeling analysis, the data from CILS were processed using the haven package [57]. The data were cleaned by converting categorical variables from haven_labelled format to numeric/factor types. Control variables relating to their self-esteem and depression were adjusted in the modeling procedures, which include gender, age, and socioeconomic status (SES) of the immigrant adolescent participants. In order to deal with a certain number of missing values, the technique of multiple imputation using the mice package was applied [58]. We used the predictive mean matching (PMM) method in the process of multiple imputation because this method is particularly useful for continuous variables due to its unbiasedly producing the predicted values for the missing variables that are closest to the regression-predicted values for the missing values from the simulated regression model randomly [59]. Further analyses were conducted based on the final imputed data; thus, no potential bias due to missing data may influence the results. Model fit was assessed by the CFI, TLI, RMSEA, and SRMR. Conventionally, cut-offs for model fit were considered adequate at CFI and TLI values higher than 0.90 and RMSEA and SRMR values less than 0.06 and 0.08, respectively [60]. In addition, we visualized the cross-lagged effects using the qgraph package for network diagrams [61]. Given this, the networks plot the strength and direction of relationships between the self-esteem and depression items across the two time periods. Green edges signify positive effects, while red edges signify negative effects; edge thickness is proportional to the standardized path coefficients.

## 3. Result

### 3.1. Descriptive Statistics

The descriptive statistics for self-esteem and depression across Time 1 and Time 2 are presented in Table 1 and Table 2. Overall, positive self-esteem items, such as “I am a person of worth”, showed a slight decrease from Time 1 (M = 1.470, SD = 0.719) to Time 2 (M = 1.307, SD = 0.581), while negative self-esteem items, such as “I certainly feel useless at times”, displayed marginal increases (Time 1: M = 2.836, SD = 1.018; Time 2: M = 2.967, SD = 0.985). Depression variables, including “Felt sad past week”, showed a slight rise from Time 1 (M = 1.685, SD = 0.831) to Time 2 (M = 1.734, SD = 0.858), indicating a minor worsening of depressive symptoms over time. Overall, the data show moderate shifts in self-esteem and depression, with a slight decline in positive self-esteem and a mild increase in depressive symptoms across the two time periods.

### 3.2. Cross-Lagged Panel Network (CLPN) Analysis

#### 3.2.1. Autoregressive Paths

**Figure 1:**
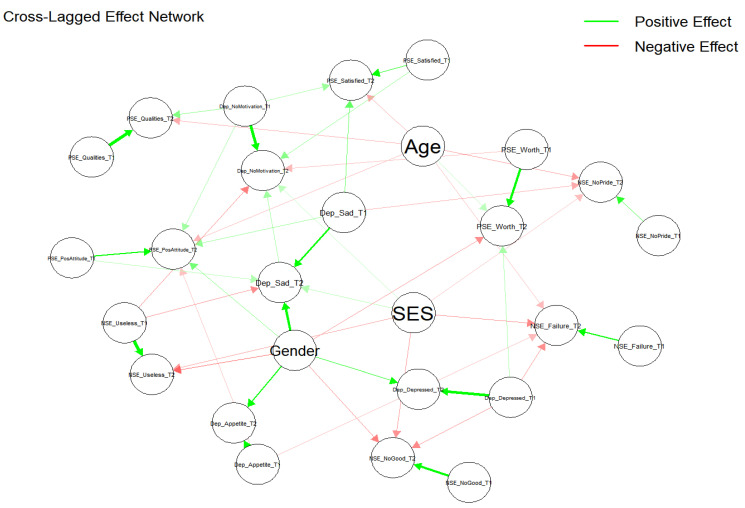
Cross-lagged effect network. Notes. This figure illustrates the significant cross-lagged effects present between variables across two time periods and demonstrates both positive and negative unidirectional effects.

The CLPN results confirm significant autoregressive paths for both self-esteem and depression among immigrant adolescents, which indicates the strong temporal stability of these psychological variables (Table 3 and Figure 1). For example, PSE_Worth at Time 1 significantly predicts PSE_Worth (β = 0.118, *p* < 0.001 ***) at Time 2. PSE_Qualities (β = 0.134, *p* < 0.001 ***), PSE_PosAttitude (β = 0.116, *p* < 0.001 ***), and PSE_Satisfied (β = 0.109, *p* < 0.001 ***) are also highly stable from Time 1 to Time 2, underlying the temporary stability of positive self-views.

The negative self-esteem dimensions also showed quite remarkable stability across times. For example, NSE_Failure at Time 1 significantly predicted NSE_Failure on Time 2 (β = 0.119, *p* < 0.001 ***), with Time 1 NSE_NoPride predicting Time 2 NSE_NoPride (β = 0.093, *p* < 0.001 ***), which shows that feelings of failure and lack of pride were consistent over time. Besides this, NSE_Useless at Time 1 was a strong predictor of Time 2 NSE_Useless (β = 0.162, *p* < 0.001 ***). NSE_NoGood_T1 at Time 1 predicted NSE_NoGood_T2 (β = 0.131, *p* < 0.001 ***).

Depressive symptoms also yielded strong temporal stability. For example, it can be seen that Dep_Sad at Time 1 predicted Dep_Sad at Time 2 (β = 0.135, *p* < 0.001 ***), whereas Dep_NoMotivation at Time 1 strongly predicted Dep_NoMotivation at Time 2 (β = 0.154, *p* < 0.001 ***). Dep_Appetite at Time 1 predicts Dep_Appetite at Time 2 (β = 0.209, *p* < 0.001 ***), and Dep_Depressed at Time 1 predicts Dep_Depressed at Time 2 (β = 0.152, *p*< 0.001 ***). These results are the temporal stability of depressive symptoms. These autoregressive effects underline the stability of both positive and negative self-perceptions and depressive symptoms over time.

#### 3.2.2. Effects of Demographic Factors

The demographic variables of immigrant adolescents’ gender, age, and SES also play significant roles in predicting their self-esteem and depression over time (Table 3). At a bivariate level, for example, male adolescents report higher levels of self-worth over time (PSE_Worth; β = −0.06, *p* < 0.001 ***) compared to female adolescents. In contrast, females report higher levels of positive attitude (PSE_PosAttitude; β = 0.086, *p* < 0.001 ***) than males. Similarly, for negative self-esteem dimensions, males are more likely to experience feeling of inadequacy (NSE_NoGood; β = −0.113, *p* < 0.001 ***) and uselessness (NSE_Useless; β = −0.141, *p* < 0.001 ***) over time than females. In the depression variable, for instance, Dep_Sad (β = 0.251, *p* < 0.001 ***), Dep_NoMotivation (β = 0.072, *p* < 0.001 ***), Dep_Depressed (β = 0.196, *p* < 0.001 ***), and Dep_Appetite_(β = 0.206, *p* < 0.001 ***) all indicate that female adolescents show higher levels of depression symptoms in sadness, lack of motivation, depression and appetite changes than male adolescents over time.

Age is significantly associated with both negative and positive self-esteem traits. On the one hand, age is inversely associated with feelings of failure (NSE_Failure; β = −0.029, *p* < 0.05 *) and unable to be proud of some achievement (NSE_NoPride; β = −0.048, *p* < 0.001 ***). Similarly, age is negatively associated with positive self-esteem traits, such as positive attitude (PSE_PosAttitude; β = −0.026, *p* < 0.05 *) and satisfaction with oneself (PSE_Satisfied; β = −0.036, *p* < 0.01 **). These results indicate that as age increases, individuals show lower levels of both positive (such as positive attitude and satisfaction with oneself) and negative traits (such as feeling of failure and no pride) from Time 1 to Time 2.

In addition, SES is positively associated with sadness (Dep_Sad; β = 0.034, *p* < 0.01 **) and lack of motivation (Dep_NoMotivation; β = 0.028, *p* < 0.05 *), which proves that family at low SES (higher SES value) contributes to higher emotional distress, including feelings of hopelessness or disengagement over time. On the other hand, SES is negatively related to perceived self-concepts of failure (NSE_Failure; β = −0.050, *p* < 0.001 ***), uselessness (NSE_Useless; β = −0.052, *p* < 0.001 ***, and worthlessness (NSE_NoGood; β = −0.055, *p* < 0.001 ***); in other words, adolescent from lower SES families (with a higher value in SES) report fewer negative self-concepts. These findings show the complex relationships between SES and adolescents’ mental health.

#### 3.2.3. Cross-Lagged Effects

PSE_PosAttitude at Time 1 positively predicts Dep_Sad at Time 2 in participants (β = 0.039, *p* < 0.05 *). Hence, individuals who perceive themselves as more positive become sadder over time. On the other hand, at Time 1, PSE_Satisfied was noted to be positively related to Dep_NoMotivation at Time 2 (β = 0.058, *p* < 0.001 ***), which proves that the higher self-satisfactory state corresponding to Time 1 tends to result in an increase in less motivation over time. PSE_Worth at Time 1 negatively contributes to depression-related symptoms motivated at Time 2 (Dep_NoMotivation: β = −0.037, *p* < 0.05 *), indicating that a greater sense of worth might offset some of the depressive symptomatology.

NSE_Useless at Time 1 negatively predicts Dep_Sad at Time 2 (β = −0.044, *p* < 0.01 **) and Dep_NoMotivation at Time 2 (β = −0.049, *p* < 0.001 ***), therefore suggesting that the feeling of uselessness at Time 1 decreases depressive symptoms, including feelings of sadness and a lack of motivation at Time 2.

Dep_Depressed at T1 predicts an increase in PSE_Worth at T2 (β = 0.025, *p* < 0.05 *), and Dep_NoMotivation at T1 predicts an increase in PSE_Qualities at T2 (β = 0.035, *p* < 0.001 ***) and PSE_Satisfied_T2 (β = 0.041, *p* < 0.01 **). Moreover, Dep_Sad at T1 positively predicts both PSE_PosAttitude (β = 0.042, *p* < 0.01 **) and PSE_Satisfied (β = 0.065, *p* <.001 ***) at T2. These results suggest that certain depressive symptoms, such as low motivation and sadness, may encourage more positive self-perceptions over time.

**Figure 2:**
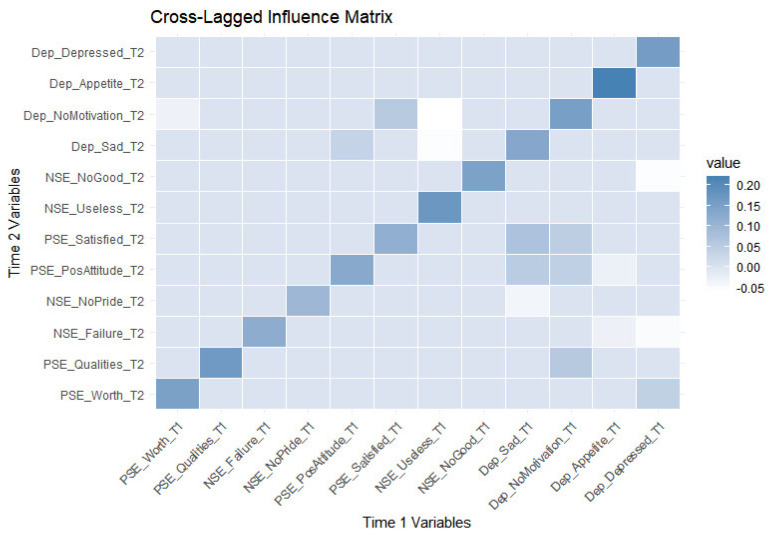
Cross-lagged influence matrix. Note: This Cross-Lagged Influence Matrix demonstrates the predictive relationships between variables across Time 1 and Time 2. Darker cells indicate stronger positive relationships, highlighting the continuity of depressive symptoms and self-esteem indicators over time, as well as their mutual influence.

Conversely, Dep_Depressed at Time 1 negatively predicts NSE_Failure at Time 2 (β = −0.045, *p* < 0.01 **) and NSE_NoGood at Time 2 (β = −0.056, *p* < 0.01 **), suggesting that depressive symptoms weaken negative self-perceptions over time, possibly as a function of emotional resignation or numbing. Dep_Appetite at T1 negatively predicts NSE_Failure at T2 (β = −0.029, *p* < 0.05 *) and PSE_PosAttitude_T2 β = −0.023, *p* < 0.05 *), indicating that appetite-related symptoms might subtly impact both negative and positive self-evaluations over time. Dep_Sad at Time 1 negatively predicts NSE_NoPride at Time 2 (β = −0.041, *p* < 0.05 *), indicating that sadness diminishes states of pride lacking over time. For more detailed information, please see Table 4 and Figure 1 and Figure 2.

#### 3.2.4. Model Fitness

The overall indices of model fit were generally good. Whereas CFI is 0.968, showing a strong fit, TLI is 0.885, showing a slightly stronger fit. For that matter, all the model fit measures were very good: the appropriately estimated RMSEA was 0.045 and SRMR was 0.048, both within acceptable ranges, thus indicating a close and exacting fit to the data overall.

## 4. Discussion

### 4.1. Autoregressive Stability of Positive and Negative Self-Esteem

It can be seen from the analysis that there is high autoregressive stability for both positive self-esteem (PSE) and negative self-esteem (NSE) indicators among immigrant adolescents over the study period. In this context, the stability highlights that individual differences in self-esteem levels remain consistent over time. However, in terms of the means reported in Table 1 and Table 2, we observe a decrease in positive self-esteem traits after three years. This variability partially agrees with the literature. As an example, Sánchez-Queija, Oliva and Parra [17] reported a linear increase in self-esteem in the period of adolescence and early adulthood, driven by positive reinforcement through relationships with peers and care from the family [1,17].

This downward trend in the self-esteem scores of immigrant adolescents suggests that they perhaps suffer from some special problems connected with cultural adjustments and social pressures of various kinds that unfavorably affect their self-assessments. The decline might reflect the additive burdens of acculturative stress and the struggle to balance identity between heritage and host culture supported through the model of ethnic identity [44,62]. Conversely, the analysis of negative self-esteem indicators reveals a different pattern. The stability of negative self-esteem variables was similarly significant. The means also show an increase in negative self-esteem traits over time. This curve reflects a very disturbing pattern and trend for adolescent immigrants negotiating identities. These feelings might be born from experiences of discrimination or social exclusion that can be extreme in immigrant communities, hence further enhancing feelings of inadequacy and failure [45,63].

Depressive symptoms demonstrated autoregressive stability over time, with early feelings of sadness, lack of motivation, poor appetite, and depression consistently predicting similar experiences at later time points. This indicates a strong temporal persistence of depressive symptoms, highlighting the enduring nature of these emotional and physical states across time [64]. The persistence of these depressive symptoms, especially the sadness and lack of motivation, serves to frame a very clear perspective on how long-standing these symptoms will be if they remain untreated and for how long emotional troubles could persist. Comparisons of means between Time 1 and Time 2 show slight increases for feelings of sadness and lack of motivation, an indication that an initial degree of sadness and low motivation may intensify under continued stress, whereas poor appetite shows a slight decrease, possibly reflecting situational adaptation [3].

The diverging trends between positive self-esteem (PSE) and negative self-esteem (NSE) suggest that while the self-concept of adolescents is resistant to changes in general, over time the quality of one’s self-concept may deteriorate. This finding supports theories that self-esteem develops under the impact of internal and external factors, with social contexts and peer relations playing a significant role [2,11]. Given the increase in negative self-esteem and decline in positive self-esteem, targeted interventions should emphasize positive regard, especially during these developmentally sensitive years [12]. Supportive programs aimed at resilience strengthening by nurturing their cultural identity become necessary in the context of immigrant adolescents [65].

Therefore, the high autoregressive stability of both positive self-esteem and negative self-esteem indicators observed among immigrant adolescents’ signals, without interventions, that individuals’ differences in self-esteem may persist into the future. This persistence makes the timing of interventions all the more imperative in order to disrupt negative trajectories and solidify positive developmental pathways. Policymakers may be able to invest in culturally responsive mentoring programs to match immigrant youths with mentors of similar backgrounds to help them through their social and academic struggles [62], thereby disrupting negative self-esteem stability. It is also at this point that educators equally play an important role regarding culturally responsive curricula, which affirm diverse identities and create a welcoming classroom environment for immigrants [66]. Personalized learning plans [67] and supportive relationships between teachers and students [68] can provide protective factors against a long pattern of negative self-esteem. Students with higher self-esteem could be provided with opportunities for leadership and skill-building in order to build their resilience and reinforce positive self-esteem. Community-based interventions [69] may buffer stability in negative self-esteem even more through the building of social support networks. Peer support programs [35] and, culturally responsive family workshops [36] may allow adolescents to feel less isolated and inadequate and may thus reduce the continuity of their negative self-esteem. Indeed, mental health resources, like the availability of counseling services, help to create a healthy self-concept and facilitate the reduction in acculturative stress for adolescents [34]. Linked efforts by policymakers, educators, and community leaders can temper these negative trends in self-esteem observed among immigrant adolescents while fostering well-being and resilience.

#### Autoregressive Effects on Age, Gender, and Socioeconomic

Gender shows notable associations with several self-esteem and depressive indicators, underscoring gendered patterns in self-concept and emotional experiences. For example, gender is negatively associated with a feeling of self-worth (PSE_Worth), suggesting that women report lower levels of self-worth than men. This agrees with previous research indicating that girls in adolescence tend to be more sensitive to negative self-concept [70], perhaps due to increased social pressure put upon them and as an effect of greater sensitivity to social evaluation in this phase [11]. Additionally, gender is positively associated with all depressive symptoms, such as sadness (Dep_Sad), depression (Dep_Depressed), no appetite (Dep_Appetite), and lack of motivation (Dep_NoMotivation), indicating that females report higher levels of depressive related feelings than males. These findings are also consistent with the literature, which shows that adolescent girls are more susceptible to internalizing symptoms of sadness and depression [71] arising from concurrent biological and psychosocial risks that enhance vulnerability to emotional stressors [72]. These gender differences suggest that targeted interventions focused on self-concept and emotional support may be particularly salient for female adolescents who are at greater risk for negative self-perceptions and depressive symptoms, for example, gender-sensitive counseling [73], to help girls cope with distress and to develop self-confidence in domains viewed typically as men’s fields.

It also shows significant associations with several dimensions of self-esteem. For example, age is negatively associated with good qualities (PSE_Qualities) and positive attitude (PSE_PosAttitude), which means that the older the adolescents, the lower they perceive and regard themselves as less positive about their being. This decline in positive self-esteem during adolescence may be related to the increased challenges and struggles with identity that generally accompany adolescence for immigrant adolescents who may be struggling with additional layers of acculturative stress and identity conflicts [11,74]. Older adolescents may become more sensitive to social comparison and to the judgments of others than younger ones, in ways that feed into declines in self-esteem while adolescents are struggling to manage dual cultural identities [42]. If so, these trends suggest that the encouragement of positive self-esteem during middle and late adolescence may serve as a buffer for such pressures, which seem to be more intense as adolescents approach adulthood. Therefore, in developing appropriate programs for age effects, emphasis should lie on acquiring positive self-esteem features for age improvement, through means of programs such as mentorship classes so as to help teenagers tackle self-criticism character and develop resilience around negative self-concepts [15,43].

Socio-economic status appears to be linked with self-esteem and depressive symptoms in a very complex way, which might suggest that adolescents from families of lower socio-economic status face specific difficulties which threaten their psychological well-being. The SES is negatively associated with feelings of failure (NSE_Failure), feeling no good_(NSE_NoGood), feelings of no pride (NSE_NoPride), and also feelings of useless (NSE_Useless). This would imply that adolescents from lower SES families (higher SES values) actually report fewer negative self-esteem traits. This result might indicate very specific pressures associated with adolescents from more wealthy families [75], such as increased expectations, performance anxiety, or social comparison. These findings provide evidence for the complex and sometimes contradictory pathways through which SES influences the mental health of adolescents. Meanwhile, SES is positively related to depressive symptoms of adolescents, such that the higher the values of SES (lower socio-economic status, e.g., poverty), the higher the sadness (Dep_Sad) and lack of motivation (Dep_NoMotivation). These findings indicate that the economic and societial challenges by low-SES families were contribute significantly to increased emotional distress [76], including feelings of hopelessness and disengagement. Such stressors may weaken adolescents’ ability to cope with economic hardship [76], limited support, and social stigma, thereby impairing their emotion regulation skills. Since emotional regulation is a key factor in a child’s resilience to depressive or negative mood symptoms, these difficulties can increase the risk of emotional vulnerability. Therefore, low-SES families not only need support in the form of financial aid but also in the form of mental health resources [21,45] and other extracurricular opportunities available in the community that ease economic burdens outcomes and provide venues for immigrant youth to develop a structured sense of self-esteem [76].

### 4.2. Differential Cross-Lagged Effects of Positive and Negative Self-Esteem on Depression

Cross-lagged effects show the clear ways in which positive and negative self-esteem indicators differently predict depressive symptoms over time. While these positive self-esteem variables such as self-satisfied (PSE_Satisfied) are usually hypothesized to be protective factors against depressive symptoms [21,36]. However, self-satisfaction (PSE_Satisfied) at Time 1 positively predicts increased motivation-related depressive symptoms (Dep_NoMotivation) at Time 2. A positive attitude (PSE_PosAttitude) at Time 1 predicts an increase in sadness (Dep_Sad) at Time 2. One possible explanation is that, while an overall positive orientation toward the self is beneficial for most adolescents’ mental health, it may interact with certain contextual or exogenous factors that would make the self-attributed positivity effect for these adolescent immigrants more diverse [45]. For instance, an initially high quantity might create unrealistic expectations, leading to disappointments or failures when facing cultural and social challenges [19] that could eventually heighten feelings of sadness over time. This is supported by theories that note acculturative stress and social thresholds have the capability to test the resilience of the immigrant youth, who may have optimism to begin with but later face external stressors or discrimination [77].

Moreover, in the second block of tests, self-worth (PSE_Worth) at Time 1 negatively contributes to the depression-related symptom of no motivation (Dep_NoMotivation) at Time 2. Which indicates that a greater sense of worth might offset some of these deterrents or causes (i.e., low motivation or fatigue) in depressive symptomatology. This may be an indication that when people feel valuable, it might be because they can sustain a sense of purpose or personal efficacy in circumstances that are challenging in nature. This indicates that a stable sense of self-esteem [17,78], as reflected in self-worth (PSE_Worth), could act as a buffering and protective factor against depressive symptoms caused by motivational dysregulation because it strengthens one’s belief in one’s self-worth and ability to remain engaged regardless of adversity [13].

In addition, although negative self-esteem variables like feeling useless at times (NSE_Useless) are typically expected to predict increases in depressive symptoms [79], this study shows an unexpected inverse relationship. Specifically, feeling useless (NSE_Useless) at Time 1 is associated with lower levels of sadness (Dep_Sad) and motivation-related depressive symptoms (Dep_NoMotivation) at Time 2. What this negative association implies is that states of “uselessness” earlier in time do not predict increased depressive symptoms later but instead are associated with declines in sadness and low motivation. One possible explanation might be operative in this finding of an inverse association is that contaminating perceptions of “uselessness” may mobilize adaptive or compensatory mechanisms whereby adolescents seek support or develop ways of coping to improve their emotional states over time. Such an assumption derives some support from views emanating from the perspective of stress inoculation theory [80,81], which stated that limited exposure to certain stressors may result in stimulating resilience and adaptive responses, both of which can reduce vulnerability to depressive symptoms.

Another meaning might be that social or environmental support systems [36] moderate this. Immigrant adolescents who early in their immigration experience feelings of uselessness might mobilize resources from their family or community and thus access emotional and social resources dampening subsequent depressive symptoms. Alternatively, some cultural backgrounds focus more on communal values or the resilience of the group, and this assists these youth in coping with negative self-assessments to transform these into positive emotional regulation strategies [77].

The findings from this study are supported by the nuanced relationships of self-esteem indicators with depressive symptoms, which call for evidence-based and targeted interventions among immigrant adolescents. For positive self-esteem traits, interventions should, therefore, focus on enabling adolescents to develop realistic self-appraisals and manage their expectations. Cognitive-behavioral therapy (CBT) programs [82], especially those that emphasize the management of expectations and cognitive restructuring, could help adolescents reappraise overly optimistic self-views and prepare them for disappointments that may emanate from cultural and social challenges. In addition, mindfulness-based stress reduction (MBSR) interventions [83] have been used to reduce emotional dissonance from external stressors and improve adaptive emotional regulation.

For negative self-esteem indicators, such as NSE_Useless, which show unexpected inverse relations with depressive symptoms, Interventions should aim at utilizing the adaptive potential of these perceptions. Programs aimed at strength-based approaches and techniques [84] for building resilience could help adolescents to transform feelings of uselessness into constructive coping efforts. Peer support groups [35] and community-based initiatives [69], such as family workshops [36], could provide additional scaffolding by fostering environments where adolescents feel understood and supported. Evidence exists that supports the idea that social and cultural support systems, in particular those emanating from communal values, can help immigrant adolescents reframe negative self-assessments into opportunities for emotional growth and regulation [85].

Finally, culturally tailored interventions addressing acculturative stress and identity negotiation become particularly important for immigrant adolescents facing dual pressures from heritage and host cultures. Programs like culturally responsive mentoring [77] or workshops on identity development [19] may be helpful in guiding adolescents in the development of a bicultural identity and in navigating the challenges of cultural adjustment. It is necessary to develop integrated efforts at the level of schools [68], families [69], and community organizations [69] in order to foster resilience and reduce depressive symptoms by taking into consideration the particular needs of this population.

### 4.3. Reciprocal Effects: The Influence of Depression Symptoms on Self-Esteem Traits

Depressive symptoms at Time 1 further affect both the positive and negative self-esteem indicators at Time 2, domonstrating a reversed relation between these constructs. Now, these bidirectional paths explain the way the experience of depression molds self-esteem over time in immigrant adolescents, who probably experience unique cultural and social situations.

It appears that some of the depressive symptoms at T1 positively predict some facets of positive self-esteem at T2. For instance, feeling depressed (Dep_Depressed) at T1 predicts an increase in self-worth (PSE_Worth) at T2, and a lack of motivation (Dep_NoMotivation) at T1 predicts an increase in good qualities (PSE_Qualities) at T2. Moreover, sadness (Dep_Sad) at T1 positively predicts both positive attitude (PSE_PosAttitude) and self-satisfaction (PSE_Satisfied) at T2. This unexpected result appears to suggest that some adolescents may actually react to depressive symptoms through compensatory mechanisms or adaptive strategies in the pursuit of positive self-esteem [86]. These compensatory mechanisms will facilitate the reaffirmation of self-worth and positive self-perceptions among the immigrant youth, usually beset with complex identity and adjustment challenges [1,74]. This is also in accordance with the theories of resilience and cognitive reappraisal, in which individuals experiencing negative emotional arousal develop increased positive self-views in order to counterbalance negative emotion and protect themselves against further deterioration [87,88].

Conversely, some depressive symptoms at T1 are associated with reductions in negative self-esteem indicators at T2. For example, the lack of appetite (Dep_Appetite) at T1 negatively predicts feelings of failure (NSE_Failure) at T2, and sadness (Dep_Sad) at T1 is linked to a decrease in lack of pride (NSE_NoPride) at T2. Additionally, depression (Dep_Depressed) at T1 negatively predicts both feelings of failure (NSE_Failure) and worthlessness (NSE_NoGood) at T2. These inverse associations may reflect the fact that depressive symptoms are succeeded over time by the efforts of adolescents to disconnect from self-critical or failure-relevant beliefs. Such a pattern would be in line with an adaptive process in which adolescents, in response to depressive symptoms, attempt to reduce negative self-evaluations as a means of shielding themselves from further emotional distress. Findings of this nature are compatible only with the cognitive reappraisal models, which involve reframing or disengaging from self-critical thoughts and serving to reduce the psychological impact of negative experiences [89].

## 5. Conclusions

The findings of the current study give emphasis to the critical importance of a multidimensional approach when applying mental health interventions, in particular at health treatment facilities. Specifically, these findings emphasize the need for interventions, considering the unique challenges for immigrant adolescents. It is important to develop programs for the positive and negative dimensions of self-esteem for this group [10], taking into consideration key demographic factors such as gender and SES. Programs imbued with resilience [88], such as building strategies and culturally responsive social support systems [15], have particular relevance when considering the interplay of self-esteem and depressive symptoms.

Both the stability and variability of self-esteem and its complex linkage with depression are instructive and potentially useful in guiding how various health care practices, educational systems, social work strategies, and public policy can better support mental health throughout the course of life [21,36]. For example, school-based interventions [85] that include cognitive reappraisal techniques [89], such as mindfulness-based stress reduction (MBSR) [83], may help adolescents manage unreal self-perceptions and reduce depressive symptoms. Moreover, community-based programs [69] may focus on peer support and family involvement, providing critical scaffolding for adolescents to navigate acculturative stress.

These findings indicate that, from a health point of view, mental health treatments at the preventive and consistent levels are highly significant, especially among vulnerable groups such as immigrant adolescents and lower SES individuals. Efforts should be channeled into building self-esteem and diminishing negative self-concepts since this might dramatically lower the risk of depression and, subsequently, lead to an improved quality of life. With the current rise in the global burden of depression, the inclusion of self-esteem enhancement in mental health treatment programs, particularly for immigrant adolescents, might be a cost-effective strategy in ensuring a reduction in the impact of depressive disorders among individuals and health agencies globally in the long term [9].

The current study has a variety of strengths that complement the existing scholarship in terms of the relationship between self-esteem and major depressive disorder, particularly in the context of immigrant adolescents. First, the use of the CLPN model enables the investigation of the bidirectional and time-varying relationship between self-esteem and depression in a way that is both innovative and sophisticated. Such a dynamic model takes into consideration the complex and reciprocal nature of the variables studied [49], further developing the field from previous models that assumed the unidirectional nature of the relations. Indeed, the positive and negative dimensions of self-esteem explored within the framework of this study achieve a deeper look into self-perception [10,23]. This is an important distinction, as the recent literature has suggested that positive and negative self-esteem may exert differential effects on mental health outcomes [10]. With the inclusion of demographic variables like gender and SES, the present study has greater external validity and targets immigrant adolescents who face a number of unique challenges. For instance, it has been established that intervention strategies aimed at this group should maximize the strengths from positive self-esteem attributes, such as self-worth, in acting as a buffer against depressive symptoms, while minimizing the possible risk associated with self-satisfaction or attitude, which is positive yet combined with unrealistic expectations. These insights could have very important implications for the design of culturally appropriate interventions that address the dual pressures of both acculturative stress [34] and self-concept development [74] within immigrant populations. For instance, interventions in building resilience or community-based support systems [15] will be fitting for their cultural and social contexts as immigrant adolescents, thus allowing a wider and more effective method of conducting mental health care [45].

Despite these many strengths, several limitations existed in this study. Reliance on self-report measures opens the potential biases for social desirability bias or inaccurate self-assessment, which may affect the validity of findings [90]. Even though self-reporting is a common method in psychological research, objective assessments of self-esteem and depression in future studies would provide advantages over self-reported information. Moreover, the sample used in the current study consists only of immigrant adolescents; therefore, generalizations across other age groups, cultural contexts, and non-immigrant populations could be limited. The unique challenges of immigrant populations, such as the influence of acculturation stress or discrimination, may serve to modify the relationship between self-esteem and depression differently than in other groups [34]. Future studies could investigate the interrelation of self-esteem and depression in various populations, including adults and members of different cultures. Another consideration is the time frame of the data used within this study, which stem from 1992 to 1995. As much as it can provide valuable information about immigrant populations during that time, one must consider changes in societal and technological contexts since then. For example, pervasive access to the internet and social media along with mobile technologies (e.g., smartphones) [91] over the past several years have changed society and may impact how contemporary youth experience the self and mental health. Although this analysis drew upon data from the 1990s, many of the issues reflected for immigrant adolescents within that time continue to be valid. Therefore, further investigations about the present relationship between self-esteem and depression can build upon our findings. The decision to use data from this time period was perhaps influenced by its availability, but it also offers an unparalleled opportunity to study immigrant populations in the context of the socio-political conditions of the time. While modern technological changes are an important consideration, the core dynamics between self-esteem and depression, particularly in immigrant populations, may be consistent over time [91]. Nevertheless, future research should test these findings against more recent data to determine whether the dynamics of self-esteem and depression have changed in light of evolving societal conditions. Additionally, future research could have a longitudinal design with more timelines, which would allow for a more comprehensive understanding of how self-esteem and depression interact over time [9]. Studies with longer timelines would help account for potential temporal changes in these psychological variables and provide insights into their long-term relationships.

In sum, interventions targeting these bidirectional dynamics may thus be considered by health care workers and policymakers in further support of the adolescents who are immigrants. Interventions on emotional regulation or cognitive reappraisal and resilience building, for example, can be employed to facilitate adolescents’ reinterpretation of depressive symptoms in a light that maintains self-views or furthers their positivity [69,85,89]. Resilience-oriented mentoring programs could use the matching of immigrant youths with mentors from similar cultural backgrounds who can act as role models for dealing with adversity while protecting self-worth [32,69,88]. Teachers also play a significant role in developing positive self-esteem through culturally responsive curricula that affirm identities and might therefore strengthen feelings of immigrant adolescents’ self-worth as a protective force against depressive symptoms [85]. Systems of community support, such as peer and family support groups, can also provide the emotional and social resources that may enable adolescents to redefine negative self-evaluation and create a resilient self-concept [69]. Access to culturally sensitive mental health counseling is important; for one thing, counselors trained in cognitive and resilience-based techniques can help immigrant adolescents cope with depressive symptoms while minimizing the impact on their self-esteem [15,32,69]. This would most definitely ensure the basic improvement of well-being across the populace while also having a preventive effect on the prevalence of depression. In fact, such programs would allow public health initiatives to foster positive self-esteem and more effectively address the mental health challenges face by society as a whole.

## Figures and Tables

**Table 1:** Descriptive statistics for self-esteem measuring items.

Items	Time 1 Node Abbreviation	Time 2 Node Abbreviation	Mean _T1	SD_T1	Mean_T2	SD_T2
RESD	Time 1	Time 2				
I am a person of worth	PSE_Worth_T1	PSE_Worth_T2	1.470	0.719	1.307	0.581
I have a number of good qualities	PSE_Qualities_T1	PSE_Qualities_T2	1.386	0.613	1.260	0.505
I’m inclined to feel I’m a failure	NSE_Failure_T1	NSE_Failure_T2	3.495	0.814	3.500	0.804
I do not have much to be proud of	NSE_NoPride_T1	NSE_NoPride_T2	3.396	0.927	3.442	0.893
I take a positive attitude toward myself	PSE_PosAttitude_T1	PSE_PosAttitude_T2	1.575	0.764	1.459	0.693
I am satisfied with myself	PSE_Satisfied_T1	PSE_Satisfied_T2	1.599	0.785	1.584	0.758
I certainly feel useless at times	NSE_Useless_T1	NSE_Useless_T2	2.836	1.018	2.967	0.985
At times I think I am no good at all	NSE_NoGood_T1	NSE_NoGood_T2	3.086	1.055	3.217	0.972

**Table 2:** Descriptive Statistics for Depression Measuring Items.

Items	Time-1 Node Abbreviation	Tme-2 Node Abbreviation	**Mean_T1**	**SD_T1**	**Mean_T2**	**SD_T2**
CES-D	TIME 1	TIME2				
Felt sad past week	Dep_Sad_T1	Dep_Sad_T2	1.685	0.831	1.734	0.858
Couldn’t get going past week	Dep_NoMotivation_T1	Dep_NoMotivation_T2	1.676	0.821	1.750	0.834
Didn‘t feel like eating past week	Dep_Appetite_T1	Dep_Appetite_T2	1.574	0.842	1.525	0.798
I felt depressed past week	Dep_Depressed_T1	Dep_Depressed_T2	1.686	0.911	1.652	0.886

**Table 3:** Autoregressive paths in cross-lagged model.

LHS	RHS	Est.	SE	z	*p*-Value	Std. All
**PSE_Worth_T2**	PSE_Worth_T1	0.118	0.009	12.431	***	0.148
**PSE_Worth_T2**	Age	0.020	0.009	2.190	*	0.030
**PSE_Worth_T2**	Gender	−0.060	0.016	−3.689	***	−0.052
**PSE_Qualities_T2**	PSE_Qualities_T1	0.134	0.009	14.790	***	0.165
**PSE_Qualities_T2**	Age	−0.023	0.008	−2.851	**	−0.039
**NSE_Failure_T2**	NSE_Failure_T1	0.119	0.012	9.834	***	0.122
**NSE_Failure_T2**	Age	−0.029	0.013	−2.265	*	−0.031
**NSE_Failure_T2**	SES	−0.050	0.013	−3.946	***	−0.054
**NSE_NoPride_T2**	NSE_NoPride_T1	0.093	0.012	7.948	***	0.097
**NSE_NoPride_T2**	Age	−0.048	0.014	−3.406	***	−0.047
**NSE_NoPride_T2**	SES	−0.030	0.014	−2.182	*	−0.030
**PSE_PosAttitude_T2**	PSE_PosAttitude_T1	0.116	0.010	11.623	***	0.129
**PSE_PosAttitude_T2**	Age	−0.026	0.011	−2.440	*	−0.033
**PSE_PosAttitude_T2**	Gender	0.086	0.019	4.533	***	0.063
**PSE_Satisfied_T2**	PSE_Satisfied_T1	0.109	0.011	10.189	***	0.115
**PSE_Satisfied_T2**	Age	−0.036	0.012	−3.040	**	−0.042
**NSE_Useless_T2**	NSE_Useless_T1	0.162	0.011	15.097	***	0.170
**NSE_Useless_T2**	Gender	−0.141	0.027	−5.276	***	−0.073
**NSE_Useless_T2**	SES	−0.052	0.015	−3.440	***	−0.047
**NSE_NoGood_T2**	NSE_NoGood_T1	0.131	0.010	12.805	***	0.145
**NSE_NoGood_T2**	Gender	−0.113	0.027	−4.243	***	−0.059
**NSE_NoGood_T2**	SES	−0.055	0.015	−3.666	***	−0.050
**Dep_Sad_T2**	Dep_Sad_T1	0.135	0.011	12.027	***	0.133
**Dep_Sad_T2**	Gender	0.251	0.023	10.895	***	0.148
**Dep_Sad_T2**	SES	0.034	0.013	2.627	**	0.036
**Dep_NoMotivation_T2**	Dep_NoMotivation_T1	0.154	0.013	12.218	***	0.153
**Dep_NoMotivation_T2**	Gender	0.072	0.023	3.210	**	0.044
**Dep_NoMotivation_T2**	SES	0.028	0.013	2.161	*	0.030
**Dep_Appetite_T2**	Dep_Appetite_T1	0.209	0.012	17.321	***	0.221
**Dep_Appetite_T2**	Gender	0.206	0.021	9.624	***	0.130
**Dep_Depressed_T2**	Dep_Depressed_T1	0.152	0.011	14.247	***	0.158
**Dep_Depressed_T2**	Gender	0.196	0.024	8.224	***	0.112

Notes. * *p* < 0.05, ** *p* < 0.01, *** *p* < 0.001. **LHS**: Left-Hand Side (dependent or predicted variable in each path). **RHS**: Right-Hand Side (independent or predictor variable in each path). **Est.**: Unstandardized path estimate, indicating the strength of association between varia-bles. **SE**: Standard Error, showing the variability of the path estimate. **z**: Z-value for testing the path estimate against zero. ***p*-Value**: Probability value indicating statistical significance. **Std.All**: Standardized path estimate

**Table 4:** Cross-lagged effects in the cross-lagged model.

LHS	RHS	Est.	SE	z	*p*-Value	Std. All
**Dep_Sad_T2**	PSE_PosAttitude_T1	0.039	0.017	2.252	*	0.036
**Dep_Sad_T2**	NSE_Useless_T1	−0.044	0.013	−3.284	**	−0.053
**Dep_NoMotivation_T2**	PSE_Worth_T1	−0.037	0.017	−2.215	*	−0.032
**Dep_NoMotivation_T2**	PSE_Satisfied_T1	0.058	0.017	3.434	**	0.055
**Dep_NoMotivation_T2**	NSE_Useless_T1	−0.049	0.013	−3.611	***	−0.060
**PSE_Worth_T2**	Dep_Depressed_T1	0.025	0.012	2.188	*	0.040
**PSE_Qualities_T2**	Dep_NoMotivation_T1	0.035	0.009	3.810	***	0.058
**NSE_Failure_T2**	Dep_Appetite_T1	−0.029	0.014	−2.122	*	−0.031
**NSE_Failure_T2**	Dep_Depressed_T1	−0.045	0.016	−2.809	**	−0.052
**NSE_NoPride_T2**	Dep_Sad_T1	−0.041	0.019	−2.148	*	−0.039
**PSE_PosAttitude_T2**	Dep_Sad_T1	0.042	0.014	2.895	**	0.051
**PSE_PosAttitude_T2**	Dep_NoMotivation_T1	0.035	0.012	2.903	**	0.042
**PSE_PosAttitude_T2**	Dep_Appetite_T1	−0.023	0.011	−2.044	*	−0.029
**PSE_Satisfied_T2**	Dep_Sad_T1	0.065	0.016	4.137	***	0.072
**PSE_Satisfied_T2**	Dep_NoMotivation_T1	0.041	0.013	3.101	**	0.045
**NSE_NoGood_T2**	Dep_Depressed_T1	−0.056	0.018	−3.079	**	−0.054

Notes. * *p* < 0.05, ** *p* < 0.01, *** *p* < 0.001.

## Data Availability

The data presented in this study are openly available in the Inter-university Consortium for Political and Social Research, which is accessible at https://www.icpsr.umich.edu/web/ICPSR/studies/20520 (accessed on 20 September 2024).
